# The Role of Tympanic Membrane Retractions in Cholesteatoma Pathogenesis

**DOI:** 10.1155/2018/9817123

**Published:** 2018-02-21

**Authors:** Letícia Petersen Schmidt Rosito, Neil Sperling, Adriane Ribeiro Teixeira, Fábio André Selaimen, Sady Selaimen da Costa

**Affiliations:** ^1^Hospital of Clinics of Porto Alegre and Department of Ophthalmology and Otorhinolaryngology, Faculty of Medicine, Federal University of Rio Grande do Sul, Rua Ramiro Barcelos 2400, Porto Alegre, RS, Brazil; ^2^Weill Cornell Medical College, New York, NY, USA; ^3^Hospital de Clínicas de Porto Alegre and Universidade Federal do Rio Grande do Sul, Department of Health and Human Communication, Rua Ramiro Barcelos 2777, Room 315, Anexo I da Saúde, Porto Alegre, RS, Brazil; ^4^Hospital de Clínicas de Porto Alegre, Rua Ramiro Barcelos 2350, Porto Alegre, RS, Brazil

## Abstract

**Objective:**

To analyze the contralateral ear (CLE) of patients with cholesteatoma and to correlate the cholesteatoma growth pattern in the affected ear with the findings in the CLE.

**Methods:**

Videotoscopy of both ears in 432 patients with cholesteatomas classified as posterior epitympanic (PEC), posterior mesotympanic (PMC), two routes, or undetermined. Tympanic membrane (TM) retractions were classified by location and severity and TM perforations according to signs of previous TM retraction.

**Results:**

TM retraction was the most prevalent alteration in the CLE (42.6%). Cholesteatoma was observed in 17.4%. In patients with PEC, the retraction in the CLE was more frequent in the PF (66.7%) than in the PT (1.4%), and in those with two-route cholesteatoma, the retraction in the CLE most frequently involved both the PT and PF (65.6%; *p* < 0.0001).

**Conclusion:**

Our results confirm the essential role of TM retraction at least in the earlier phases of cholesteatoma pathogenesis.

## 1. Introduction

Although several centuries have passed since its first description by Duverney in 1689, the pathogenesis of acquired middle ear (ME) cholesteatoma is still debated [1]. At present, four major theories can be defined as follows: metaplasia (transformation of the inflamed middle ear mucosa into keratinized squamous epithelium), migration (ingrowth of the squamous epithelium through a peripheral perforation), invagination (progressive retraction of the tympanic membrane [TM]). and papillary proliferation (infection leading to proliferation of epithelial cones in the basal layers of the TM) [2]. Among these theories, invagination is one of the most operational ones. However, clinical studies carried out by Sudhoff and Tos [2] and Sadé et al. [3, 4] have failed to validate the transition of TM retractions to cholesteatoma, since several patients were lost to follow-up, and the cumulative incidence of cholesteatoma was too small. Thus, alternative designs to test this hypothesis clinically are needed. Our objective was to analyze TM retractions and cholesteatomas in the contralateral ear (CLE) of patients with acquired cholesteatoma in a search of new clues to explain more comprehensively the natural history of this disease.

## 2. Materials and Methods

We included 432 consecutive patients with acquired cholesteatoma between August 2000 and December 2015. Exclusion criterion was a history of any ear surgery except tympanostomy.

Patients' detailed clinical histories were recorded, both ears were examined using a fiber-optic otoendoscope, and images were acquired. Images were then independently reviewed and blinded, so that changes in the CLE were described without any awareness of the primary ear presentation.

For data analysis, the primary ears were defined as either having cholesteatoma or being more symptomatic. We classified the cholesteatomas based on their growth pattern as (a) posterior epitympanic (PEC), (b) posterior mesotympanic (PMC), (c) two routes, or (d) undetermined [5].

TM retraction was classified by location and severity according to a modified version of the classification by Sadé et al. [3, 4]. When retraction of both the pars flaccida (PF) and the pars tensa (PT) was observed, the severity of these two retractions was compared and the following two groups constituted (i) retraction of the PF only or both the PF and the PT, with PF retraction being more severe, and (ii) retraction of the PT only or both the PT and the PF, with PT retraction being more severe.

TM perforations were classified into two groups according to the signs of previous TM retraction: medialization of the manubrium of the malleus, remnant tympanum adhered to the ossicular chain, remnant tympanum adhered to the promontory, and ossicular chain erosion. Those with at least two signs were classified as “outside-in.” All the others were classified as “inside-out.”

The authors assert that all procedures contributing to this work comply with the ethical standards of the relevant national and institutional guidelines on human experimentation (Group Research and Graduate Studies Department, protocol number 14920) and with the Helsinki Declaration of 1975, as revised in 2008.

Statistical analysis was performed using chi-square and Fisher's exact test. All tests were two-sided. Statistical significance was set at* p* ≤ 0.05.

## 3. Results and Analysis

The mean (SD) patient age was 33.3 (19.9) years and 233 patients (53.9%) were women. PMC and PEC were present in the primary ears of 146 (33.8%) and 145 (33.6%) patients, respectively. Two-route cholesteatomas were observed in 68 (15.7%) patients and undetermined cholesteatomas in 73 (16.9%) main ears.

Only 147 (34.0%) of the CLEs were considered normal. TM retraction was the most frequent change (*n* = 184, 42.6%). Cholesteatoma was observed in 75 (17.4%) and TM perforation in 26 (6.0%) CLEs.

**Figure 1:**
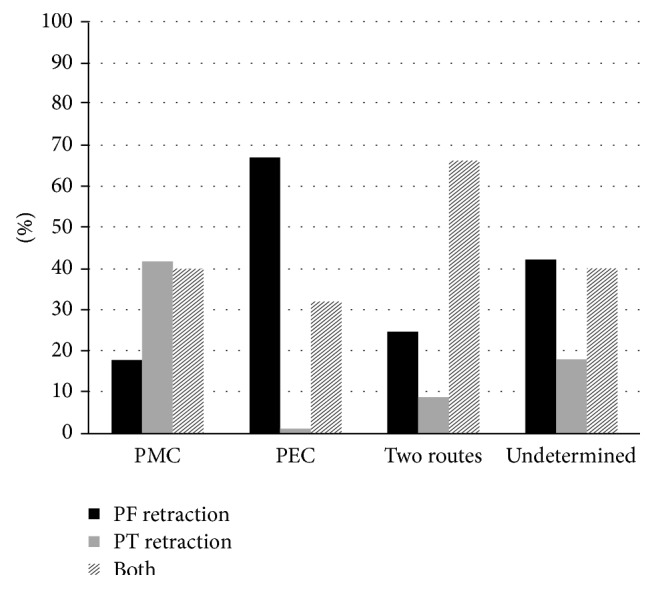
Overall prevalence of tympanic membrane retraction in the contralateral ear according to the cholesteatoma growth pattern in the primary ear. PMC: posterior mesotympanic cholesteatoma. PEC: posterior epitympanic cholesteatoma. Two routes: two-route cholesteatoma. Undetermined: undetermined cholesteatoma. PF retraction: pars flaccida retraction. PT retraction: pars tensa retraction. Both: pars flaccida and pars tensa retraction.

Analyzing only the 184 patients with moderate and severe TM retraction in the CLE, we observed that when the primary ear presented PMC, retraction was more prevalent in the PT than in the PF. In patients with PEC, the retraction in the CLE was more frequent in the PF than in the PT, while, in those with two-route cholesteatomas, the retraction in the CLE was most frequently observed in both the PT and the PF (*p* < 0.0001), as shown in Figure 1.

**Figure 2:**
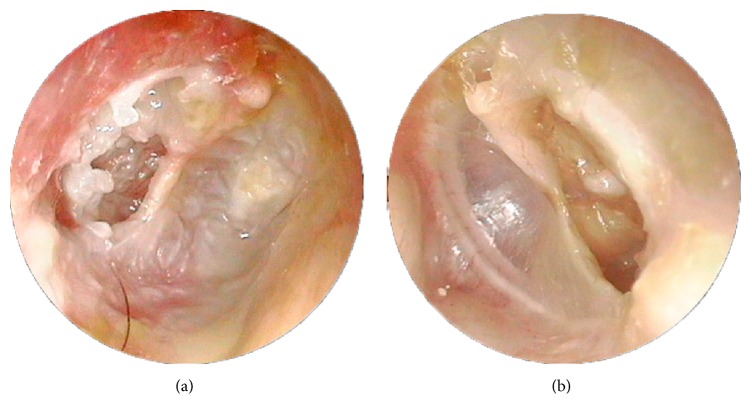
Videotoscopy of posterior mesotympanic cholesteatoma and severe* pars tensa* retraction: both ears of the same patient. (a) Posterior mesotympanic cholesteatoma. (b) Severe* pars tensa* retraction.

When TM retraction was analyzed according to the two groups, it was found mainly in the PF in 60 patients (92.3%) with PEC and mainly in the PT in 46 patients (78.0%) with PMC (*p* < 0.0001). This association is illustrated in Figure 2.

**Figure 3:**
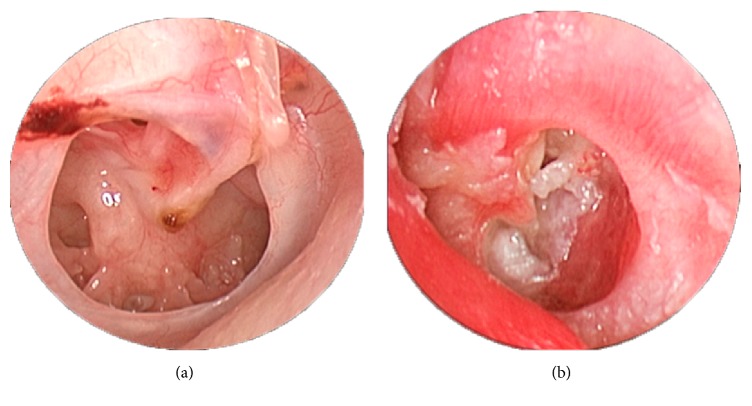
Outside-in tympanic membrane perforation in the contralateral ear and posterior mesotympanic cholesteatoma in the left ear of the same patient. (a) Outside-in tympanic membrane perforation in the contralateral ear. (b) Posterior mesotympanic cholesteatoma in the left ear.

Among the 26 patients with TM perforation in the CLE, perforation was “inside-out” in 12 (46.2%) patients and “outside-in” in 14 (53.8%) patients, as demonstrated in Figure 3.

**Figure 4:**
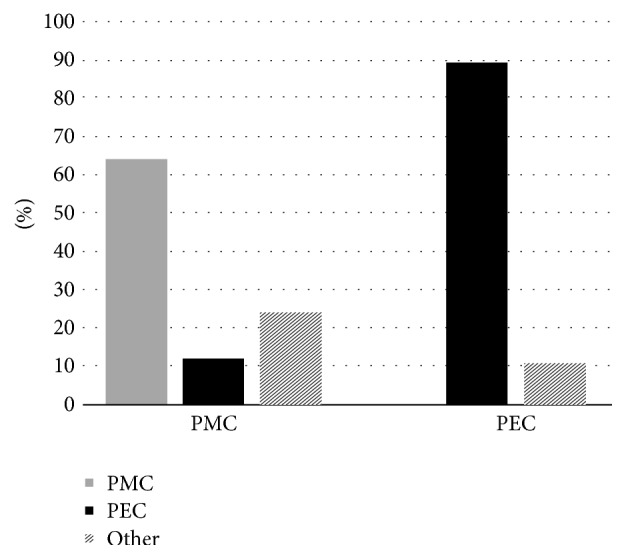
Comparison of the cholesteatoma growth patterns in the primary and contralateral ears. PMC: posterior mesotympanic cholesteatoma. PEC: posterior epitympanic cholesteatoma. Other: other types of cholesteatoma.

**Figure 5:**
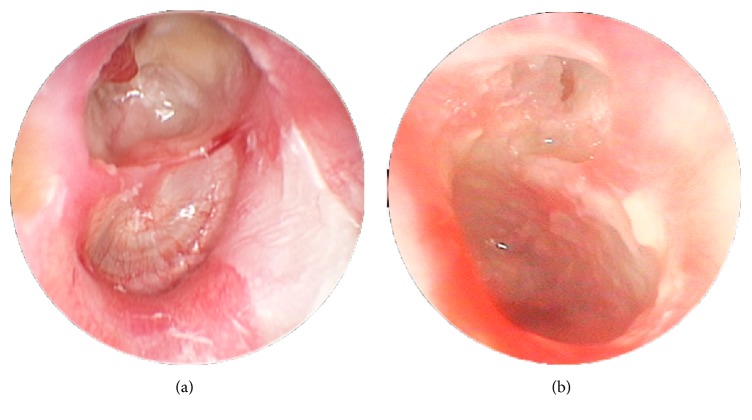
Posterior epitympanic cholesteatoma in the right ear and posterior epitympanic cholesteatoma in the left ear of the same patient. (a) Posterior epitympanic cholesteatoma in the right ear. (b) Posterior epitympanic cholesteatoma in the left ear.

Of the 50 patients with PEC and PMC in primary ears and cholesteatoma in the CLE, we observed that 81% with PEC presented with PEC in the CLE. Further, when PMC was observed in the primary ear, the same growth pattern was observed in 55.2% of the CLEs, as demonstrated in Figure 4 (*p* < 0.0001). This association is illustrated in Figure 5.

## 4. Discussion

Since 2008, we have been studying the pathogenesis of chronic otitis media (COM) by examining the CLE [6]. Our observations have systematically shown a high prevalence of alterations in the CLE in clinical [6], histopathological [7], functional [8], and radiological [9] studies. Moreover, the frequency of alterations in the CLE was even higher in patients with cholesteatoma [6, 10]. It has been pointed out that the anatomy could be similar between the main and contralateral ear but Eustachian tube function and ventilation routes towards the epitympanum through the isthmus may vary due to several factors. Still, our hypothesis for this amazing similitude between the ears is that they share a common embryogenesis and are both subjected to the same environmental triggers. This fact also holds true to explain that the vast majority of otitis media with effusion cases in children are also bilateral.

Analyzing only those with alterations in the CLE, we observed in the present study that 95.8% of the patients presented with retraction or signs of previous retraction (outside-in perforations), or progression of these retractions (cholesteatoma) in the CLE. Interestingly, our results show a strong association between growth patterns of cholesteatomas in the main ear and the location of TM retractions in the CLE. Therefore, it seems plausible to infer that these retractions retrospectively represent the initial phases of cholesteatoma formation in the main ear. Jackler et al. [11] proposed that the vast majority of acquired cholesteatomas arise when a pouch of the TM draws into the attic and/or posterior mesotympanum [10]. Our findings also suggest that TM retraction is an event that precedes cholesteatoma formation.

Mechanisms responsible for progressive TM retraction are still debated. Eustachian tube (ET) dysfunction resulting in impaired middle ear ventilation has been indicated as an important factor. Cauterization of the ET resulted in retraction of the PF and cholesteatoma in 75% of the gerbils treated [12]. Paradoxically, patent ET can also result in ME alterations [13, 14]. ME inflammation may also explain the increased gas loss rate [15–17]. Whatever the causative mechanism, negative pressure seems to play at least an initial role in TM retractions.

Another question to be addressed is why the retractions develop preferentially in the PF and the posterosuperior aspects of the PT. We postulate that the site of the obstruction is related to the creation of hypoventilated microspots. According to Bhide [18], decreased ME pressure leads to a medial displacement of the TM and the handle of the malleus toward the promontory. The posterosuperior quadrant appears to be structurally more vulnerable, and a triangular microspot is then created and bounded by the handle of the malleus, a bony bar extending from the subiculum, the dome of the promontory, and the corresponding annulus.

In relation to PF retraction, the tympanic isthmus seems to have a crucial role. It is the main route of drainage and aeration of the attic chambers and can be occluded by mucosal edema, thick mucus plugs, or retraction of the posterior part of the PT [19–21]. Prussak space aeration route opens directly into the mesotympanum via the posterior pouch. Histopathological studies have demonstrated that although the dimensions of the posterior pouch vary among individuals, they are bilaterally symmetrical [22]. We believe that, once created, these microspots may become stable through tight fibrous adhesions between the inner mucosal layer of the TM and the mucoperiosteum of the ossicles and the ME (which may become the precursor of the future cholesteatoma perimatrix), regardless of the reestablishment of ME ventilation. As suggested by Jackler [23], although a ME vacuum could initiate TM retraction, it cannot credibly be the sustaining force for progressive growth of the cholesteatoma pouch. Epitympanum, aditus, and antrum become blocked early in the course of the disease and subsequently fill with mucous and/or inflammatory tissues; creation of a vacuum due to gas reabsorption is impossible under these circumstances.

Whether the TM retraction per se is enough to cause cholesteatoma formation is still a matter of contention. We believe that other factors that can disrupt the stability of the retraction are essential. Sudhoff and Tos [2] proposed a four-step concept for the pathogenesis of cholesteatoma that combines the retraction and proliferation theories. On the other hand, the theory of mucosal traction proposed by Jackler is based on the premise that the squamous pouch is drawn inward by the interaction of opposing motile surfaces of middle ear mucosa [11]. The only point of convergence of these theories is that TM retractions were almost universally implied in the first stages of cholesteatoma formation.

In conclusion, when bilateral, cholesteatomas tend to follow the same growth pattern in both ears. Furthermore, severe and moderate tympanic membrane retractions, the most frequent alterations found in the CLE, tend to occur in the same site of primary ear cholesteatoma. Finally, our results also endorse the essential role of TM retraction in the cholesteatoma pathogenesis.
